# Genetic and virulence characteristics of a *Raoultella planticola* isolate resistant to carbapenem and tigecycline

**DOI:** 10.1038/s41598-022-07778-0

**Published:** 2022-03-09

**Authors:** Ying Li, Yichuan Qiu, Yan Gao, Wenbi Chen, Chengwen Li, Xiaoyi Dai, Luhua Zhang

**Affiliations:** The School of Basic Medical Science and Public Center of Experimental Technology, Southwest Medical University, Luzhou, 646000 Sichuan Province China

**Keywords:** Antimicrobials, Bacterial genomics, Bacterial pathogenesis, Microbiology, Water microbiology

## Abstract

*Raoultella planticola* is an emerging pathogen causing several infections in humans, and its roles in the propagation of antibiotic resistance genes (ARGs) remain uncharacterized. In this study, a carbapenem and tigecycline-resistant *R. planticola* isolate was recovered from hospital sewage. It carried nine plasmids, bearing 30 ARGs, including one *bla*_KPC-2_ and two *bla*_NDM-1_. It also contained a plasmid-borne efflux pump gene cluster, *tmexCD1-toprJ*, conferring resistance to tigecycline. Analysis of plasmid sequences revealed that both *bla*_NDM-1_-carrying plasmids were highly similar to those recovered from humans, reinforcing the close relatedness of environmental and clinical isolates. We also identified that plasmid bearing *bla*_NDM-1_ or *tmexCD1-toprJ1* was transferable, and can be stabilized in the host bacteria, indicating that the *R. planticola* isolate has a considerable potential in the dissemination of ARGs. Besides, we found that this isolate could produce biofilm and was virulent in a *Galleria mellonella* infection model. In conclusion, our study shows the convergence of virulence and multidrug resistance in a *R. planticola* isolate. This potentially virulent superbug may disseminate into its receiving rivers, and finally to humans through cross-contamination during recreation activities or daily use of water, which poses a risk to public health.

## Introduction

The development of antibiotics provided an effective treatment for bacterial infections, and markedly reduced the health and economic burden associated with infectious diseases^1^. However, the rapid emergence and prevalence of antibiotic resistance has posed a great threat to the human and animal health at the global level^2^. Especially, the widespread of carbapenem- and colistin-resistant Enterobacterales represents a major challenge due to limited treatment options^3,4^. In this scenario, tigecycline, classified as a critically important antimicrobial by the WHO (World Health Organization), was considered as one of the last therapeutic options against infections caused by these bacteria^5^. Unfortunately, the clinical potential of tigecycline has been significantly compromised by the recent emergence of novel mobile *tet(X)* orthologs that confer high-level tigecycline resistance^5,6^. In addition, plasmid-borne *tmexCD-toprJ* homologs, encoding Resistance-Nodulation-Division (RND) family multidrug efflux pumps that confer tigecycline resistance, were also identified in Enterobacterales^7,8^. The emerging transferable tigecycline resistance determinants represent a new threat to global public health.

Antibiotic resistance is more than a clinical problem. The complex and close interactions between humans, animals, and environments have contributed to the propagation and spread of antibiotic-resistant bacteria (ARB) across all community sectors^9^. Addressing these issues, a holistic approach known as the One Health concept was proposed at the animal-human-environment interface to tackle the expanding antibiotic resistance and reduce the risk of infectious diseases globally^10^. The aquatic environment, especially hospital sewage, which is a mixing pool of antibiotic residues and ARB from nosocomial settings and excrement of patients, serves as a vast reservoir of ARGs^11^. With high concentrations of antibiotics and resistant organisms, hospital sewage also serves as a hotspot for horizontal gene transfer, enabling the exchange of ARGs between bacterial communities, ultimately generating multidrug-resistant (MDR) organisms^12^. Feng et al. reported the isolation of an *Acinetobacter johnsonii* strain harboring nine plasmids and encoding NDM-1 and OXA-58 carbapenemases from hospital sewage in 2010 in China^13^. Besides, hospital sewage also provides an ideal platform to generate new mobile elements, such as hybrid plasmids and novel transposons, via active genetic events, which facilitates a more flexible dissemination of antimicrobial resistance among bacteria^14^.

*Raoultella planticola*, belonging to the Enterobacteriaceae family, is a Gram-negative, non-motile, rod-shaped anaerobic bacterium^15^. It was first found by Freney et al. in 1984 in a patient with sepsis, and was originally classified as the *Klebsiella* spp., while it was reclassified as a member of the *Raoultella* genus based on 16S rRNA and *rpoB* gene analysis in 2001^16–18^. This bacterium was primarily considered as harmless, environmental organism that mostly grows in soil and water^16^. However, in recent years it was identified as an emerging virulent pathogen due to its close association with many reports of severe human infections, including liver abscesses^19^, cholangitis^20^, pancreatitis^21^, conjunctivitis^22^, acutecholecystitis^23^ and urinary tract infections^24^*.* Though, factors contributing to the pathogenesis of *Raoultella spp.* remains largely uncharacterized. Besides, a number of MDR *R. planticola* isolates harboring carbapenemase genes, such as *bla*_KPC-2_^25^, *bla*_OXA-48_^26^, *bla*_NDM-1_^27^ and *bla*_IMP-8_^28^, have been reported in humans, implying a possibly underestimated role of this bacterial species in the spread of antimicrobial resistance. The combination of MDR and hypervirulence would significantly limit options for treating severe infections, causing a particular threat for human health.

In this study, we report a carbapenem- and tigecycline-resistant *R. planticola* isolate harboring nine plasmids from hospital sewage. We aim to elucidate the transmission mechanisms of carbapenem and tigecycline resistance determinants by analyzing the plasmids carrying them. Also, we investigated the pathogenicity of the MDR *R. planticola* isolate, to our knowledge, have not been described previously.

## Materials and methods

### Bacterial isolation

*R. planticola* SCLZS62 was isolated during a study for the presence of carbapenem-resistant Enterobacteriaceae strains in hospital sewage. 5 ml of water sample was collected from the influx mainstream of hospital sewage at the affiliated hospital of Southwest Medical University, Luzhou in western China, in November 2019. As described in our previous study^29^, bacterial cells were concentrated by centrifugation at 5000*g* for 5 min. The sediment was resuspended in sterile 0.9% NaCl solution and plated onto MacConkey agar containing meropenem (2 μg/ml) and incubated for 24 h at 37 ℃. Pink colonies with various morphologies were picked and repeatedly streaked on new MacConkey agar plates to obtain pure isolates. Initial species identification was performed by PCR amplifying of 16S rRNA gene and Sanger sequencing^30^. The presence of the acquired carbapenemase genes, *bla*_KPC_, *bla*_NDM_, *bla*_OXA-48_, *bla*_OXA-58_, *bla*_VIM,_ and *bla*_IMP_ was screened via PCR assays using primers as previously described^29^.

### Antimicrobial susceptibility testing

The minimum inhibitory concentrations (MICs) of amikacin, gentamicin, colistin, meropenem, imipenem, cefoxitin, chloramphenicol, ciprofloxacin, cefotaxime and tigecycline against the target strains were determined using the broth microdilution method according to the Clinical and Laboratory Standards Institute (CLSI) guidelines (CLSI, 2017). Breakpoints for colistin and tigecycline were defined by European Committee on Antimicrobial Susceptibility testing (EUCAST) guidelines (http://www.eucast.org/clinical_breakpoints/). *Escherichia coli* ATCC 25922 was used as a quality control for MIC determination. All antibiotics used in this study were obtained from yuanye Bio-Technology Co. (Shanghai, China).

### Conjugation assay

Conjugation experiments were performed using broth-based methods. The azide-resistant *E. coli* strain J53 was used as the recipient, and transconjugants were selected on Luria–Bertani (LB) agar plates containing 4 μg/ml meropenem or 4 μg/ml tigecycline plus 150 μg/ml sodium azide. Sixty-four and twenty-three transconjugants were randomly selected from meropenem- and tigecycline- containing plates for PCR assay, respectively. The presence of plasmids p1_SCLZS62, p2_SCLZS62, p3_SCLZS62, p4_SCLZS62, p5_SCLZS62, p6_SCLZS62, p7_SCLZS62, p8_SCLZS62 and p9_SCLZS62 in transconjugants was confirmed by PCR using the primers p1-F/R, p2-F/R, p3-F/R, p4-F/R, p5-F/R, p6-F/R, p7-F/R, p8-F/R and p9-F/R in Table S1.

### Genome sequencing and analysis

Genomic DNA of *R. planticola* SCLZS62 was extracted and purified using the QIAamp DNA Mini Kit (Qiagen). The generated DNA was sheared with an average size of 10 kb and submitted for whole genome sequencing using a PacBio RSII sequencer (Pacific Biosciences, Menlo Park, USA). Meanwhile, the genomic DNA was sequenced on a HiSeq 2000 sequencer (Illumina, San Diego, CA, USA), using a paired-end library with an insert size of 150 bp. The de novo assembly of the PacBio reads was carried out with the Link v5.0.1. BWA-MEM was employed for mapping Illumina reads over the PacBio-generated contigs to correct the assembled contigs^31^. Library construction and sequencing was carried out at Beijing Novogene Bioinformatics Technology Co. Ltd. Annotation was carried out using Prokka^32^ and BLASTP searches against the UniProtKB/Swiss-Prot database. The species identification was carried out by average nucleotide identity (ANI) analysis with the online software JSpeciesWS (http://jspecies.ribohost.com/jspeciesws/#analyse). Digital DNA-DNA hybridization (dDDH) values were calculated using GGDC 3.0 server (http://ggdc.dsmz.de/distcalc2.php) by means of genome-to-genome sequence comparison^33^. Plasmid incompatibility types were analysed using the PlasmidFinder tool (95%, minimum threshold for identity; 60%, minimum coverage)^34^, and antimicrobial resistance genes were predicted using ResFinder (90%, minimum threshold for identity; 60%, minimum coverage)^35^. Insertion elements (ISs) and integrons were predicted using ISfinder^36^ and INTEGRALL^37^. The presence of virulence genes was investigated by searching the virulence factor database (VFDB) with an *E* value cutoff of 0.0001^38^. The retrieved virulence genes were further screened with a cutoff of > 50% query coverage and > 75% identity. Multiple and pairwise sequence comparisons were carried out using the BRIG tool^39^. Gene organization diagrams were visualized with Inkscape 0.92.4 (https://inkscape.org/en/).

### Phylogenetic analysis

Genomes were annotated using Prokka and the generated GFF3 files were used to create a core genome alignment with Roary. Single nucleotide polymorphisms (SNPs) were obtained with snp-sites v2.3.2^40^. Based on the SNPs, A maximum-likelihood phylogenetic tree was constructed with FastTree version 2.1.10^41^. The presence of β-lactamase genes was detected by Abricate (https://github.com/tseemann/abricate). Carriage of β-lactamase genes and detail information of isolates were annotated on the tree using iTOL^42^.

### Gene cloning

DNA fragment containing *baeSR*-*tmexAB-toprM*, *tmexAB-toprM* and *baeSR*-*tmexAB* were amplified from *R. planticola* SCLZS62 using the primers F1/R1, F2/R1 and F1/R2 in Table S1. The resulting PCR product was then ligated into a cloning vector pMD19-T using a ClonExpress® II One Step Cloning Kit (Vazyme, China) to give pMD19-*baeSR*-*tmexAB-toprM*, pMD19-*tmexAB-toprM*, pMD19-*baeSR*-*tmexAB*. The recombinant vectors were then electroporated into *E. coli* DH5α and verified by PCR assays. *E. coli* DH5α containing empty pUC19 served as a negative control for antimicrobial susceptibility assay.

### Plasmid stability

The stability of p2_SCLZS62, p5_SCLZS62, p7_SCLZS62 and p8_SCLZS62 in *R. planticola* SCLZS62 was studied as previously described with little modification^7^. SCLZS62 was grown overnight in 3 ml LB (antibiotic-free) broth at 37 °C, and 3 µl overnight culture was then incubated into 3 ml fresh LB broth each day, yielding ~ 10 generations of growth per passage. The serial passage was lasted for 21 days. Every three days, cultures were serially diluted and plated on LB agar plates without antibiotics. To determine the percentage of plasmid-containing cells, ~ 100 colonies were screened on LB agar plates containing 4 μg/ml tigecycline or meropenem. The presence of p2_SCLZS62, p5_SCLZS62, p7_SCLZS62 and p8_SCLZS62 was confirmed by PCR assays using primers p2-F/R, p5-F/R, p7-F/R and p8-F/R, respectively, in Table S1. This experiment was performed in triplicate.

### In vitro growth assays

Three independent cultures of J53 and transconjugants were grown overnight and diluted to 1:100 in LB broth. Bacteria cultures were incubated while shaking at 37℃. In the total period of 12 h, the value of optical density (OD) at 600 nm (OD600) was consistently recorded at an interval of 1 h with the iMark microplate Reader (Bio-Rad).

### Biofilm formation assays

Biofilm formation assays were performed as described previously with minor changes^43^. Overnight cultures of *R. planticola* SCLZS62 was diluted 1/100 into fresh LB broth, and 200 μl of bacterial suspensions was inoculated into sterile 96-well microplates plates in triplicate. After 24 h at 37 ℃, culture supernatant was removed and the wells were washed with phosphate-buffered saline (PBS) and stained with 200 μl 0.1% (w/v) crystal violet solution at room temperature for 15 min. After that, the wells were washed with PBS for three times to remove excess stain and dry for 1 h. The bound dye was released by adding 100 ul of acetic acid (33%, v/v) and the optical density was measured at 595 nm using a microplate reader. NTUH-K2044, a hypermucoviscous and hypervirulent *Klebsiella pneumoniae* isolate belonging to the sequence type 23 and capsular type K1 from a patient with primary liver abscess^44^, and MG1655, a non-pathogenic *E. coli* K-12 strain^45^, were included as the positive and negative control strains, respectively. LB broth without any inoculation serves as a blank control. This experiment was performed in triplicate.

### *Galleria mellonella* infection assays

The virulence potential of the *R. planticola* SCLZS62 was assessed using wax moth (*Galleria mellonella*) larvae weighing 250 to 350 mg (Tianjin Huiyude Biotech Company, Tianjin, China) as described previously with minor changes^46^. Overnight culture of *R. planticola* SCLZS62 from LB agar plates was harvested and adjusted using PBS to final concentrations of 1 × 10^6^ CFU/ml, 1 × 10^7^ CFU/ml, 1 × 10^8^ CFU/ml and 1 × 10^9^ CFU/ml. Ten larvae were chosen randomly as a group, and 10 μl aliquots of bacterial suspension was injected into the last left proleg of each larvae using a 50 μl Hamilton syringe. Larvae injected with the *K. pneumoniae* NTUH-K2044 were used as hypervirulence-positive controls, the *E. coli* MG1655 as low-virulence controls, and PBS as negative controls. The larvae were then incubated at 37 °C, and the number of live larvae was counted at 12 h intervals for 3 days. In all cases, no dead larvae were observed in the PBS groups. This experiment was performed in triplicate, and a representative result of three independent experiments was used to generate survival curves.

### Statistical analysis

Data of biofilm formation assays were analyzed with a student *t* test performed in the GraphPad Prism version 8.3.0 (GraphPad Software, CA, USA). A two-way analysis of variance (ANOVA) was used to evaluate statistical significance in the bacterial growth assays. The survival rate of the *G. mellonella* was analyzed using the log-rank test. Differences were considered statistically significant at *P* < 0.05.

## Results and discussion

### Genome characterization of *R. planticola* SCLZS62

SCLZS62 was a MDR strain as it exhibited resistance to all tested antibiotics (≥ 3 classes of antimicrobial agents), except for colistin^47^ (Table 1). Whole genome sequencing showed that SCLZS62 had a chromosome of 5,490,846 bp, with a GC content of 55.88% and a total of 5185 open reading frames (ORFs). It also carried nine plasmids designated as p1_SCLZS62 to p9_SCLZS62, ranging in size from 10.5 to 334.2 kb and encoding 14 to 382 ORFs (Table 2). SCLZS62 belongs to *R. planticola* as it had 99.02% identity (89.81% query coverage) to the *R. planticola* reference strain FDAARGOS_64 by ANI analysis, and the dDDH value between them was 93.10%, both above the suggested cut-off for defining a bacterial species. In consistence with its multidrug resistance profile, SCLZS62 had 30 antibiotic resistance genes, mediating resistance to aminoglycosides (*aac*(*6′*)*-Ib3*, *aadA5*, *ant*(*2″*)*-Ia*, *armA* and *rmtC*), β-lactams (*bla*_KPC-2_, *bla*_NDM-1_, *bla*_CTX-M-14_, *bla*_PLA2a_ and *bla*_PER-1_), macrolide (*mph*(*A*), *mph*(*E*) and *msr*(*E*)), fosfomycin (*fosA* and *fosA3*), quinolones (*aac*(*6′*)*-Ib-cr*, *qnrA1* and *qnrS1*), rifampicin (*ARR-3*), sulfonamides (*sul1*), trimethoprim (*dfrA1*), and tetracycline (*tet*(*A*)). The detection of carbapenemase genes *bla*_KPC-2_ and *bla*_NDM-1_ explained the resistance to all the cephalosporin and carbapenem drugs tested. It was notable that *R. planticola* SCLZS62 contained three genes conferring resistance to carbapenems, including two *bla*_NDM-1_ and one *bla*_KPC-2_, which were carried by three different plasmids. In addition, it was observed that *R. planticola* SCLZS62 was resistant to tigecycline, and the recently characterized plasmid-mediated tigecycline resistance genes *tet(X)* variants were absent. Further analysis of the genome data showed that the newly identified efflux pump gene cluster, *tmexCD1-toprJ1*, involved in tigecycline resistance was identified on a plasmid in *R. planticola* SCLZS62, which probably contributed to the resistance of this isolate to tigecycline.

**Table 1:** MICs for SCLZS62, their transformants and the recipient strain J53.

Strains	MIC (μg/ml)									
	AMK	GEN	CST	MEM	IMP	CFT	CHL	CIP	CTX	TGC
SCLZS62	**> 512**	**512**	≤ 0.5	**512**	**512**	**> 512**	4	**8**	**> 512**	**8**
J53	4	2	≤ 0.5	≤ 0.5	≤ 0.5	8	2	≤ 0.5	≤ 0.5	≤ 0.5
J53/p2 (JM-20)	**> 512**	**512**	≤ 0.5	**128**	**128**	**512**	2	≤ 0.5	**512**	≤ 0.5
J53/p1 + p2 + p5 (JM-9)	**> 512**	**512**	≤ 0.5	**128**	**512**	**> 512**	2	≤ 0.5	**512**	≤ 0.5
J53/p2 + p5 + p7(JM-5)	**> 512**	**512**	≤ 0.5	**256**	**128**	**> 512**	4	**16**	**512**	**4**
J53/p2 + p5 + p8 (JM-52)	**512**	**32**	≤ 0.5	**64**	**64**	**> 512**	4	≤ 0.5	**256**	≤ 0.5
J53/p2 + p5 + p8 + p7(JM-63)	**256**	**16**	≤ 0.5	**128**	**128**	**> 512**	4	≤ 0.5	**256**	**8**
J53/p2 + p5 + p7(JT-4)	**> 512**	**512**	≤ 0.5	**64**	**64**	**> 512**	16	**16**	**> 512**	**8**
*E. coli* ATCC25922	4	2	≤ 0.5	≤ 0.5	≤ 0.5	8	4	≤ 0.5	≤ 0.5	≤ 0.5

Resistant MIC′s are highlighted in bold.

AMK, amikacin; GEN, gentamicin; CST, colistin; MEM, meropenem; IMP, imipenem; CFT, cefoxitin; CHL, chloramphenicol; CIP, ciprofloxacin; CTX, cefotaxime; TGC, tigecycline.

**Table 2:** Summary of the genetic features of *R. planticola* SCLZS62.

Chromosome /plasmid	Length (bp)	GC%	No. of predicted ORFs	Inc type	Drug resistance gene
Chromosome	5,490,846	55.88	5185	–	*fosA*,* bla*_*PLA2a*_,* bla*_CTX-M-14_
p1_SCLZS62	10,509	47.07	14	Col440I	
p2_SCLZS62	110,781	54.85	125	IncFII(Yp)	*rmtC*,* bla*_NDM-1_,* sul1*
p3_SCLZS62	116,031	49.82	127	IncFIB(pHCM2)	
p4_SCLZR53	125,664	52.26	144	UT	*qnrS1*
p5_SCLZS62	173,509	51.79	213	IncC	*ant(2″)-Ia*,* bla*_NDM-1_,* qnrA1*,* sul1*^*a*^
p6_SCLZS62	266,366	46.65	340	pKPC-CAV1321	
p7_SCLZS62	334,207	48.63	382	IncHI5	*aac(6′)-Ib3*,* aadA5*,* armA*,* bla*_PER-1_,* fosA3*,* mph(A)*,* mph(E)*,* msr(E)*,* aac(6′)-Ib-cr*,* qnrS1*,* ARR-3*,* sul1*^*a*^,* tet(A)*,* dfrA1*
p8_SCLZS62	42,847	49.72	62	UT	*bla*_KPC-2_
p9_SCLZS62	74,646	53.16	87	UT	

^a^Multiple copies on the plasmid.

–, not available; UT, unknown type.

### Phylogenetic analysis of *R. planticola* SCLZS62

**Figure 1:**
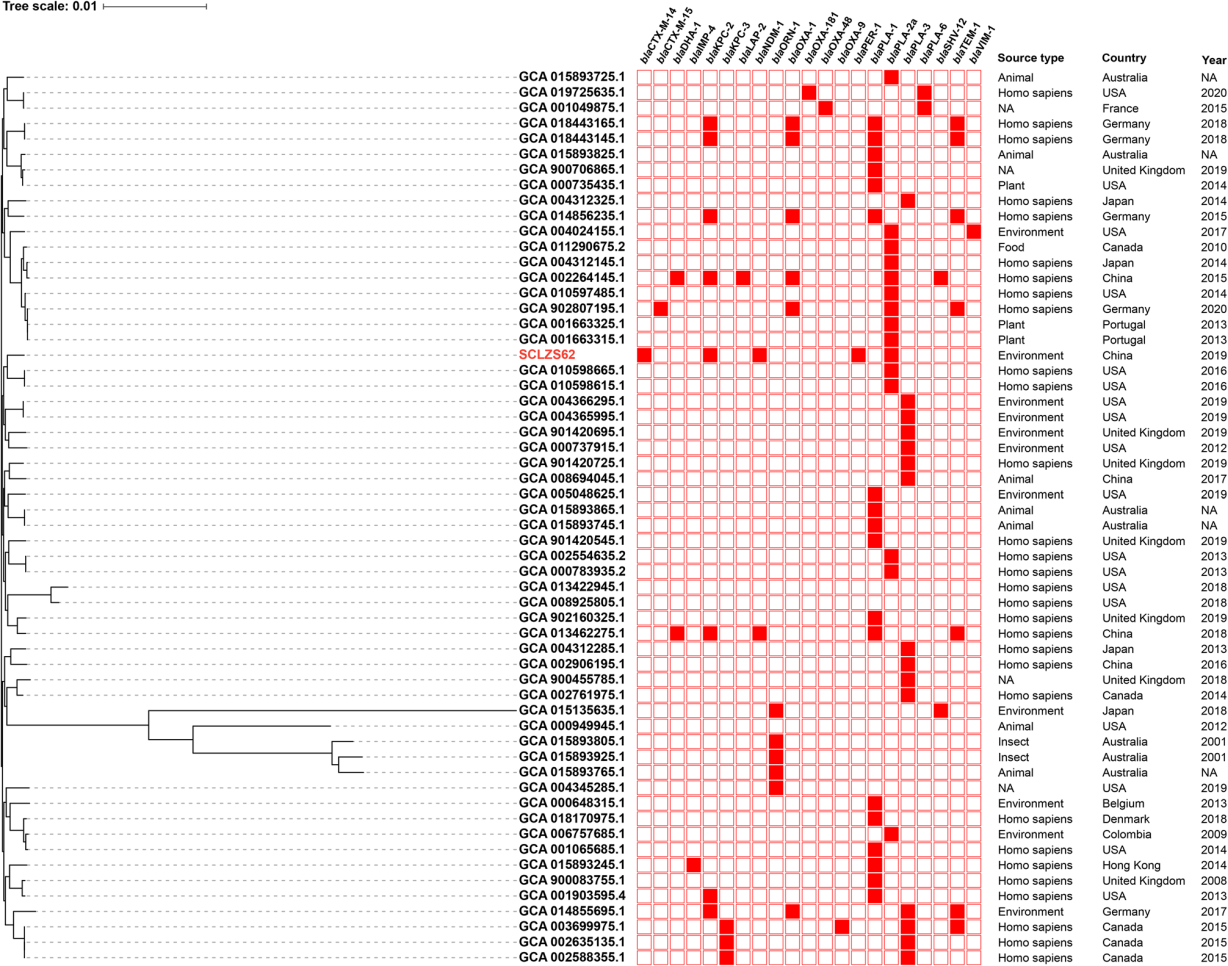
Phylogenetic tree of SCLZS62 with other 57 *R. planticola* genomes available from GenBank. The tree is based on 655 core genes with 39,331 SNPs, and the tree scale indicates substitutions per site. SCLZS62 is indicated in red. Resistance gene profiles are visualized in compliance to the tree. The annotation denotes (from left to right) isolation sources, locations, and years of strains. NA, not available. Detailed information of these *R. planticola* strains is presented in Table S2.

To determine a possible clinical relevance of SCLZS62, whole-genome sequences of 57 publicly *R. planticola* strains retrieved from GenBank (on 2021/09/02) were aligned with that of SCLZS62. A core genome-based phylogeny showed a diverse set of genomes, with a total of 655 core genes and 39,331 SNPs. These *R. planticola* isolates were mainly recovered from humans and the environment, and also found in animals, plant and insects across various countries. Phylogenetic tree showed that SCLZS62 was present in a cluster with two isolates AS012264 and AS012263 (Accession no. GCA_010598615.1 and GCA_010598665.1) from patients with lung disease in USA in 2016, with 1430 and 1428 SNPs, respectively (Fig. 1, Table S2). This result revealed a potential close clinical relevance of *R. planticola* SCLZS62. Resistance gene profiles of all *R. planticola* isolates showed a sporadical acquisitions of carbapenemase genes, with *bla*_KPC-2_ being the most frequent one, followed by *bla*_NDM-1_. SCLZS62 and another clinical isolate (GCA_013462275.1), also from China in 2018, represent the only two strains coharboring *bla*_KPC-2_ and *bla*_NDM-1_, with 1508 SNPs differences.

### Genetic features of the ***bla***_NDM-1_-harbouring plasmid p2_SCLZS62

p2_SCLZS62 is a 110,781-bp circular plasmid belonging to the IncFII(Yp) group, with a total of 125 annotated ORFs and an average GC content of 54.85%. BLAST search of the plasmid sequences against the GenBank database showed p2_SCLZS62 is highly similar (100% query coverage and > 99.9% nucleotide identity) to several *bla*_NDM_-harboring plasmids recovered from humans (Figure S1), such as pRJF866 (GenBank accession no. KF732966) from *K. pneumoniae* (China, 2014), pNDM-SCNJ07 (Accession no. MK933278) from *Enterobacter hormaechei* (China, 2019), pNDM-Ec1GN574 (Accession no. KJ812998) from *Enterobacter cloacae* (Canada, 2014), pEC4-NDM-6 (Accession no. KC887916) from *E. coli* (New Zealand, 2013), and pNDM_4TM (Accession no. MF042352) from *Serratia marcescens* (Romania, 2015). In all of these plasmids, *bla*_NDM_ was located in a ~ 6.0 kb region bracketed by two copies of Tn3-derived inverted-repeat transposable elements (TIMEs, bases 6434 to 6689 and 23,920 to 24,175 of p2_SCLZS62), which contribute to the mobilization of *bla*_NDM_. In addition to *bla*_NDM-1,_ p2_SCLZS62 also harbored resistance genes *rmtC* and *sul1*, which were located in a 11.5-kb region upstream of *bla*_NDM-1_ that was also flanked by two copies of TIMEs.

### Genetic features of the ***bla***_NDM-1_-harbouring plasmid p5_SCLZS62

**Figure 2:**
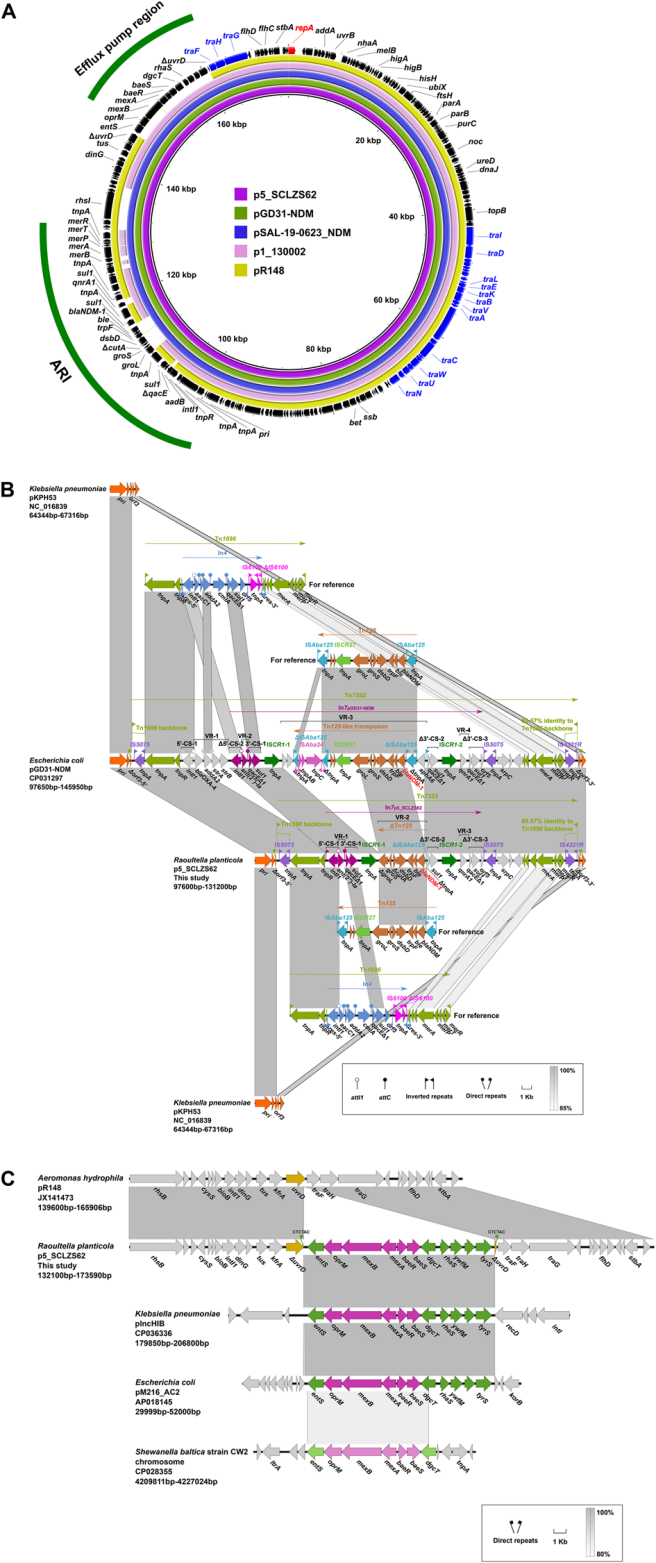
Genetic features of p5_SCLZS62. (**A**) Circular comparison of p5_SCLZS62 with type 1 IncC plasmids. The complete sequence of p5_SCLZS62 was used as the reference. Arrows on the outer ring indicate deduced ORFs and their orientations. The replication gene *repA* is highlighted in red, and genes for conjugal transfer are indicated in blue. Two accessory resistance regions (ARI and Efflux pump region) are indicated by green curves. (**B**) Organization of the ARI of p5_SCLZS62, and comparisons to related regions. Genes are denoted by arrows, and are colored based on their functional classification. Regions of > 85% homology are indicated by grey shadings. The accession numbers of Tn*1696* and Tn*125* for reference are U12338 and JN872328, respectively. Δ represents truncated genes. CS, conserved segment; VR, variable region. (C) Comparison of the efflux pump region of p5_SCLZS62 with those of closely related sequences. The *baeSR*-*tmexAB-toprM* gene cluster is highlighted in red, and remaining genes in this region are indicated in green. The gene *uvrD*, being indicated in yellow, is interrupted by the insertion of the efflux pump region, leaving 6-bp direct target repeats (CTCTAC). Other plasmid backbone genes and hypothetical genes are colored gray. Regions of > 80% homology are indicated by grey shadings.

Sequence analysis of p5_SCLZS62 indicated that it was a circular IncC-type plasmid of 173,509 bp with 213 predicted ORFs. Based on the presence or absence of *orf1832*/*orf1847*, *rhs1*/*rhs2*, i1, and i2, which are key features distinguishing between type 1 and type 2 IncC plasmids, p5_SCLZS62 was recognized as the type 1 IncC plasmid^48^. A BLAST search against the GenBank database showed that p5_SCLZS62 was almost identical (100% coverage and ≥ 99.98% identity) to several plasmids recovered from humans, including pGD31-NDM (Accession no. CP031297) in *E. coli* from Viet Nam and pSAL-19–0623 (Accession no. MN604267) in *Salmonella enterica* from Singapore (Fig. 2A).

Compared with the type 1 prototype plasmid pR148, p5_SCLZS62 shared a 128-kb backbone, but the content of antibiotic resistance island, designated ARI, was varied (Fig. 2A). It is notable that all resistance genes of p5_SCLZS62, including *ant(2″)-Ia*,* qnrA1*,* sul1* and *bla*_NDM-1_ are located in the ARI, with the structure of a novel unit transposons, designated Tn*7353*, being inserted at a site within a gene named *orf3* encoding a putative permease in the plasmid pKPHS3 (Accession no. NC_016839) from clinical *K. pneumoniae* isolate (Fig. 2B). The Tn*7353* was a derivative of Tn*1696*, with identical *tnpA-tnpR* and 89.57% nucleotide identity of the *mer* gene cluster, but differed from it mainly by insertion of a complex class 1 integron instead of In*4* in Tn*1696*. The complex class 1 integron consists of one 5′-CS, three 3′-CSs, two IS*CR1*s, and three VRs bearing resistance genes. *bla*_NDM-1_ was located in the IS*CR1*-1-linked VR-2, with the structure of ΔTn*125* consisting of Δ*groL*,* groS*, Δ*cutA*,* dsbD*,* trpF*,* ble*, *bla*_NDM-1_ and ΔIS*Aba125* in order, within In*7*_p5_SCLZS62_. According to a previous report, IS*CR27* may be involved in the initial acquisition and mobilization of *bla*_NDM-1_, and then Tn*125* was commonly associated with the wide dissemination of *bla*_NDM-1_ in bacteria^49^. The presence of IS*CR1* downstream of the truncated Tn*125* in this complex class 1 integron suggests an important role of IS*CR1* in the evolution and further movement of the *bla*_NDM-1_ gene, by mobilizing the ΔTn*125* segment in this situation. The construction of the complex class 1 integron harboring multiple resistance genes by the introduction of two IS*CR1* elements revealed significant roles of mobile elements like IS*CR1* in the creation and evolution of multidrug- and pandrug-resistant regions^47^, which poses a big challenge in fighting against antibiotic resistance. By sequence comparison, the ARI of p5_SCLZS62 showed high similarity (99% query coverage and 99.98% identity) to the corresponding region of pGD31-NDM, which was also identified as a derivative of Tn*1696* termed Tn*7352*, with some deletions: (1) A 3577-bp deletion of the cassette array, *bla*_OXA-4_-*aadA2*-*strA*-*strB*, within the class 1 integron, which probably resulted from homologous recombination via the 5′-CS of the class 1 integron; (2) two deletions at the IS*CR1*-1-associated VR-2, including a 7856-bp deletion downstream of the IS*CR1*-1 and a 1780-bp deletion downstream of *bla*_NDM-1_. We speculated that ARI of p5_SCLZS62 might have progressively evolved from the genetic structure like Tn*7352* by experiencing multiple genetic events.

In addition to the ARI, p5_SCLZS62 also contained an 15-kb region harboring a novel efflux pump gene cluster, designated *tmexA-tmexB-toprM* (“t” for transferrable^7^), when it was compared with pR148. The efflux pump region had an *ents*-*toprM-tmexB-tmexA-baeR-baeS-dgcT-rhaS-ywfM-orf-tyrS* structure, which was inserted into the *uvrD* gene, splitting it into two separate parts and meanwhile leaving 6-bp direct repeats (DRs, CTCTAC) at both ends (Fig. 2C). A BLASTn search in the GenBank database showed that the efflux pump region was also found in plasmids pIncHIB (Accession no. CP036336) in *K. pneumoniae* from India, and pM216_AC2 (Accession no. AP018145) in *E. coli* from Japan. Specially, the plasmid-borne efflux pump gene cluster *baeS-baeR-tmexAB-toprM* was found to be an 88.87% match to a chromosomal DNA fragment of *Shewanella baltica* strain CW2 (Accession no. CP028355), raising the possibility that this efflux pump gene cluster originates from certain species of *Shewanella*, such as *S. baltica.* Despite the presence of perfect DRs, there was no mobile element found in the efflux pump region. The transposition mechanism of this region requires further elucidation.

Sequence analysis showed that this novel efflux pump tMexA-tMexB-tOprM, showed 36.02%, 55.6%, 46.04% amino acid identity to the MexA, MexB, OprM encoded on the chromosome of *Pseudomonas aeruginosa*, respectively. This gene cluster *tmexAB-toprM* is adjacent to ORFs annotated as a two-component regulatory system encoded by *baeS* and *baeR*, indicating an intact gene cluster encoding an efflux pump. To validate the function of *tmexAB-toprM*, three recombinant plasmids: pMD19-*baeSR*-*tmexAB-toprM*, pMD19-*tmexAB-toprM* (missing *baeSR*), pMD19-*baeSR*-*tmexAB* (missing *toprM*) were constructed. Relative to those with the empty vector pUC-19, none of the *E. coli* DH5α strain carrying recombinant plasmid showed increase in the MICs of the tested antimicrobial agents (Table S3), suggesting that this *tmexAB-toprM* failed to function as an efflux pump system. Additional studies are needed to fully characterize the biological functions of the *tmexAB-toprM* gene cluster.

### Genetic features of plasmid p7_SCLZS62

**Figure 3:**
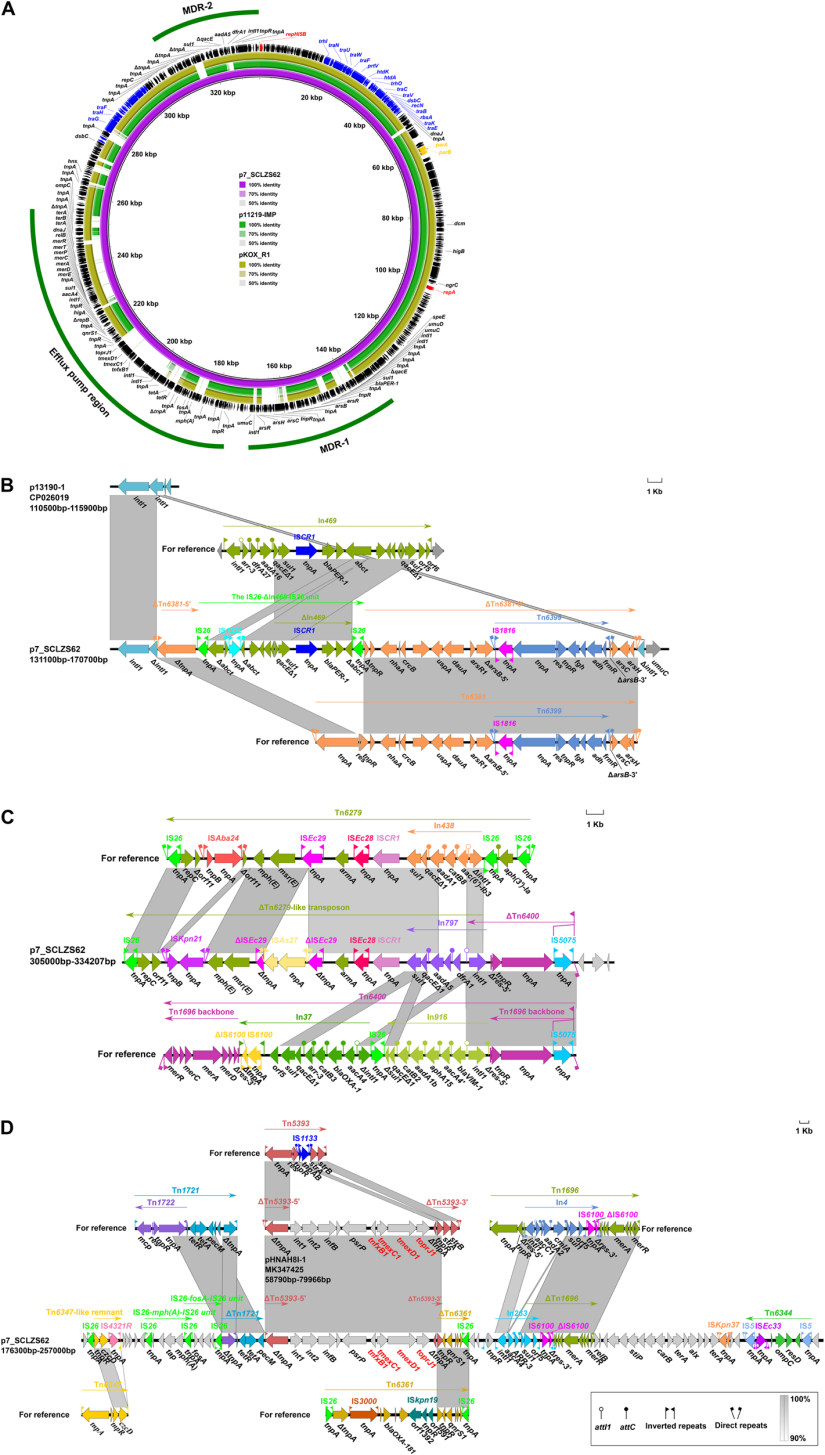
Genetic features of p7_SCLZS62. (**A**) Circular comparison of p7_SCLZS62 with IncHI5 plasmids. The complete sequence of p7_SCLZS62 served as the reference. Arrows on the outer ring indicate deduced ORFs and their orientations. The replication gene *repA* is highlighted in red, conjugal transfer genes in blue, and *parAB* for partition in yellow. Three accessory regions MDR-1, MDR-2, and Efflux pump region are indicated by green curves. (**B**) Organization of the MDR-1 of p7_SCLZS62, and comparison to related regions. The accession numbers of In*469* and Tn*6381* for reference are KP076293 and MF344566, respectively. (**C**) Organization of the MDR-2 of p7_SCLZS62, and comparison to related regions. The accession numbers of Tn*6279* and Tn*6400* for reference are KT317075 and KU318421, respectively. (**D**) Organization of the efflux pump region of p7_SCLZS62, and comparison to related regions. The accession numbers of Tn*5393*, Tn*1721*, Tn*1696*, Tn*6347*, Tn*6361* and Tn*6344* for reference are M96392, X61367, U12338, MF344562, KM660724 and MF344567, respectively. Genes are denoted by arrows, and are colored based on their functional classification. Regions of > 90% homology are indicated by grey shadings. Δ represents truncated genes.

p7_SCLZS62 has 334,207-bp circularly closed DNA sequences, and carries 541 predicted ORFs in total. p7_SCLZS62 belongs to IncHI5 group because it contains a replication gene *repHI5B,* and an additional *repFIB-*like gene. A BLASTn search against the GenBank database showed the highest 87% query coverage, and 99.93% identity to the plasmid pJNQH579-2 (Accession no. CP078148) in *Klebsiella variicola* from a clinical sputum specimen in China. Genomic comparison of p7_SCLZS62 with two IncHI5 reference plasmid pKOX_R1 (Accession no. CP003684) and p11219-IMP (Accession no.MF344561) showed that it possess conserved backbones, including *repHI5B* (together with its iterons) and *repFIB-*like gene for replication, *parAB* for partition, and two *tra* regions (*tra1* and *tra2)* for conjugal transfer^50^(Fig. 3A). This plasmid harbors numerous antibiotic resistance genes (Table 2), as well as a newly identified RND efflux pump gene cluster, *tmexCD1-toprJ1*, which confers transferable resistance to tigecycline^7^.

p7_SCLZS62 carries three accessory modules, namely the MDR-1 region, MDR-2 region, and efflux pump region. The MDR-1 region was identified as a Tn*6381* derivative, which was inserted at a site within an *intl1* gene, breaking it into two separate parts Δ*intl1*-5′ and Δ*intl1*-3′ (Fig. 3B). The Tn*6381* derivative was generated from insertion of IS*26*-ΔIn*469*-IS*26* unit into a site downstream of *tnpA*, leading to a 651-bp deletion in the Tn*3*-family core transposition module *tnpA*-*res*-*tnpR,* and a truncation of both *tnpA* and *tnpR.* The MDR-2 region consists largely of a ΔTn*6279*-like transposon, and the rest was a *tnpA*-*tnpR* module with its terminal 38-bp IRL interrupted by IS*5075* and a *res* remnant *res-5*′ (Fig. 3C). As a derivative of Tn*6279*, the ΔTn*6279*-like transposon had major modifications including an insertion of IS*Kpn21* and IS*As27*, a deletion of IS*Aba24* and the terminal IS*26*-*aph(3′)-Ia*-IS*26* unit, and a replacement of In*438* by In*797.* The efflux pump region was identified as a complex chimera structure composed of a Tn*6347*-like remnant, two IS*26*-composite transposon-like units (IS*26*-*mph(A)*-IS*26* and IS*26*-*fosA*-IS*26*), ΔTn*1721*, ΔTn*6361*, In*268*, ΔTn*1696*, Tn*6344* and the efflux pump module (Δ*tnpA-int-int-infB-psrP*-*tnfxB1-tmexC1- tmexD1-toprJ1-*Δ*tnpA- tnpR*) that was most likely to be derived from Tn*5393* (Fig. 3D). The efflux pump module in p7_SCLZS62 shared high similarity (95% coverage, 99.97% identity) to that in pHNAH8I-1, the first reported *tmexCD1-toprJ1*-harboring plasmid^7^. Unlike the *strA-strB* of Tn*5393*-3′ retained in the pHNAH8I-1, forming *tnpR-strA-strB*, a Tn*6361* remnant was located downstream of *tnpR* in p7_SCLZS62, and formed the *tnpR-qnrS1-*IS*26* structure*.* The acquisition of ΔTn*6361* in p7_SCLZS62 was most likely to result from massive recombination events between two copies of *tnpR*.

### Genetic features of the ***bla***_KPC-2_-harbouring plasmid p8_SCLZS62

**Figure 4:**
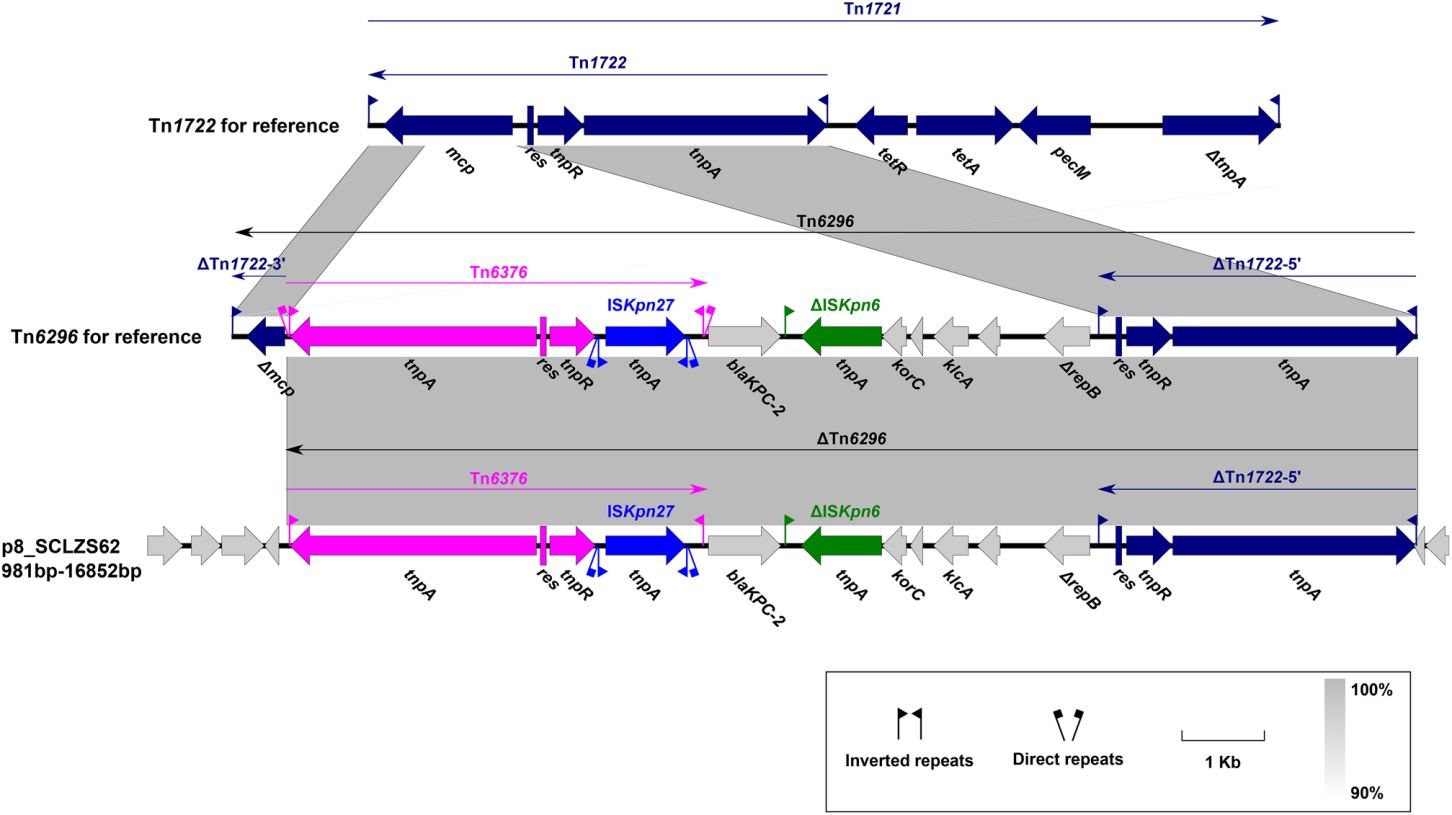
Organization of genetic context of *bla*_KPC-2_ and comparison to related regions. Genes are denoted by arrows, and are colored based on their functional classification. Regions of > 90% homology are indicated by grey shadings. The accession numbers of Tn*1721* and Tn*6296* for reference are X61367 and FJ628167, respectively. Δ indicates truncated genes.

Plasmid p8_SCLZS62 is a circular molecule of 42,847 bp with an average GC content of 49.72%, and harbors 62 predicted ORF. It could not be assigned to any known incompatibility group, and the *bla*_KPC-2_ gene was the sole resistance determinant of this plasmid. Full plasmid sequence queries of p8_SCLZS62 against the NCBI GenBank showed that it has high similarity (> 99% query coverage and > 99% nucleotide identity) to pHS062105-3 (Accession no. 023331), pCP40 (Accession no. MH328006), pP10159-3 (Accession no. MF072963) and pCKPC18-1(Accession no. CP022276), which were recovered from humans and the environment in different regions of China (Figure S2). This result reinforced the important role of the pCKPC18-1-like untypeable plasmids in the dissemination of *bla*_KPC-2_ in *Enterobacteriaceae* in China^51–53^. The backbone of these plasmids include an untypeable replication initiation gene (*repA*), *parA* and *topB* for stability, *relEB* genes for maintenance, and *tviB* for conjugation. In the accessory region, *bla*_KPC-2_ was carried by a ΔTn*6296* genetic platform, in which ΔTn*1722-3′* was lost from the prototype Tn*6296* (Fig. 4)*.*

### Plasmid transferability, stability and host fitness

**Figure 5:**
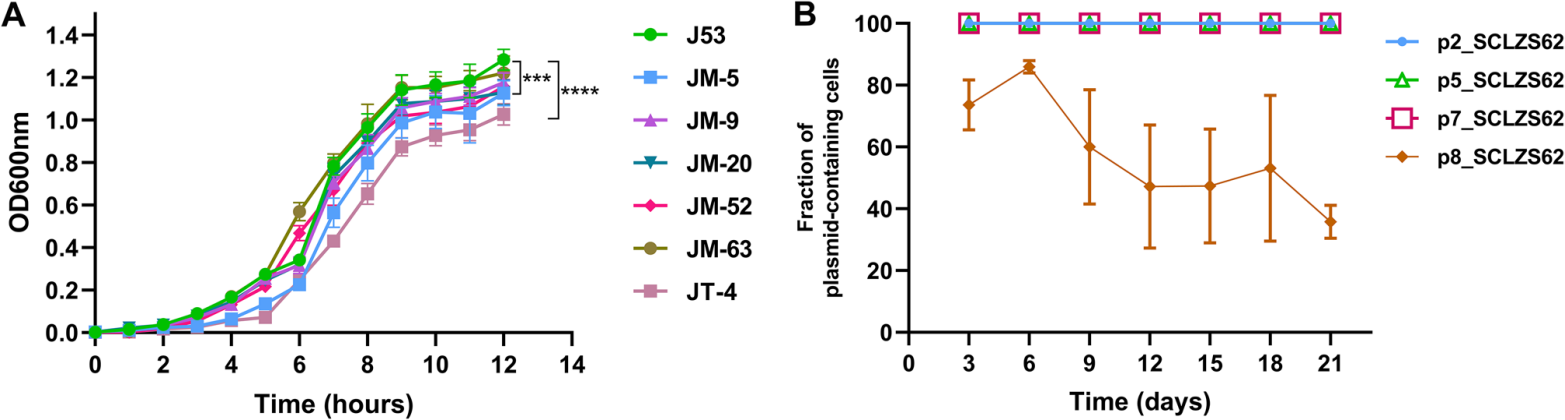
Characterization of the host fitness and stability of resistant plasmids. (**A**) Growth curves for the transconjugants and the recipient strain J53. (**B**) Stability of plasmids p2_SCLZS62, p5_SCLZS62, p7_SCLZS62 and p8_SCLZS62. Data were expressed as means ± standard deviations. Error bars show SDs, and asterisks indicate statistical significance using two-way ANOVA (***P < 0.001, ****P < 0.0001).

Conjugal transfer experiments showed that carbapenem resistance determinants could be transferred into *E. coli* J53 at the frequency of ~ 10^–6^ (transconjugant/recipient). Among the transconjugants, cells containing p5_SCLZS62 accounted for 73.44%, and p8_SCLZS62 only for 4.69%. Specially, all the transconjugants harbored p2_SCLZS62. This result indicates that these three plasmids carrying carbapenemase genes are mobilizable, with the highest transferability of p2_SCLZS62, and the least efficiency of p8_SCLZS62. MICs of carbapenem (including meropenem and imipenem) and cephalosporin (including cefoxitin and cefotaxime), of these transconjugants, were increased at least 128-fold (Table 1). In addition, p7_SCLZS62 was able to conjugate into *E. coli* J53 at a frequency of ~ 10^–8^, which is consistent with its intact set of conjugative transfer genes. Antimicrobial susceptibility analysis revealed that the acquisition of p7_SCLZS62 enabled *E. coli* J53 to become resistant to tigecycline by at least 8-fold (Table 1). To estimate the fitness cost of these resistant plasmids, we compared growth characteristics of the transconjugants harboring resistant plasmids, and the plasmid-free recipient strain J53. Results showed that transconjugants JM-5 and JT-4, which bear plasmids p2_SCLZS62, p5_SCLZS62 and p7_SCLZS62, exhibited a significant growth retardation compared to J53, indicating that the coexistence of these three plasmids confers a fitness cost on the host (Fig. 5A).

Stability assays showed that both carbapenem and tigecycline resistance determinants were stable for ≥ 21 d (~ 210 generations) in the *R. planticola* SCLZS62 in antibiotic-free medium. Among the carbapenem-resistant colonies, plasmids p2_SCLZS62 and p5_SCLZS62 were stably maintained with 100% retention, while p8_SCLZS62 was gradually lost during serial passage with 35.76 ± 5.35% retention after 21 days (Fig. 5B). The transferability and highly stability of plasmids containing *bla*_NDM-1_ or efflux pump gene cluster *tmexCD1-toprJ1* reinforced the idea that MDR strains like *R. planticola* SCLZS62 in the aquatic environment serve as a reservoir of ARGs, which has serious public health implications.

### Virulence characteristics of *R. planticola* SCLZS62

**Figure 6:**
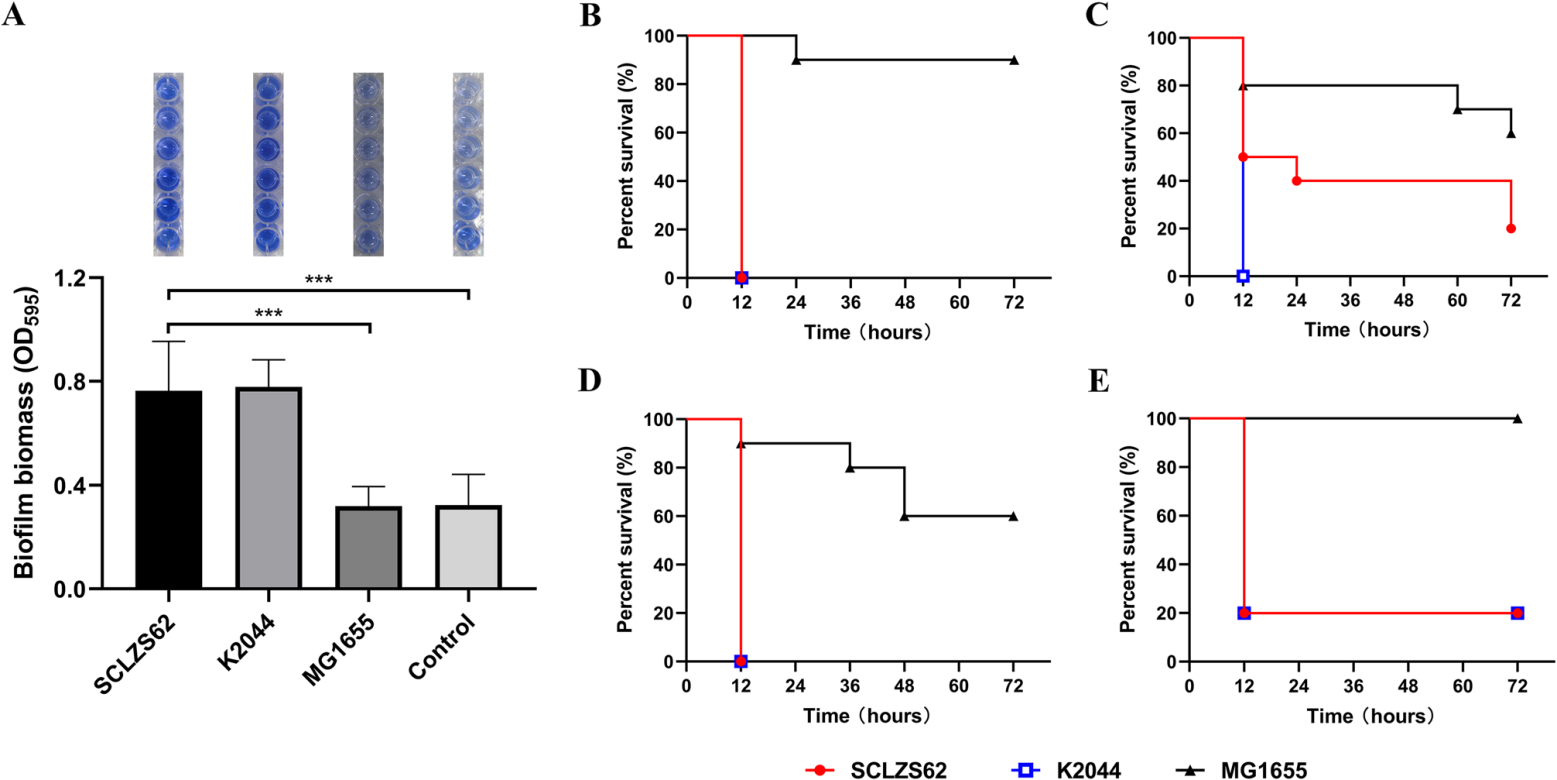
Virulence characteristics of *R. planticola* SCLZS62. (**A**) Biofilm formation assays of *R. planticola* SCLZS62, *K. pneumoniae* NTUH-K2044 (positive control) and *E. coli* MG1655 (negative control). Biofilms in the 96-well plates, which were stained using crystal violet and released by acetic acid, were shown above the barchart. Quantitative analysis of biofilms was performed by measuring the optical density at 595 nm using a microplate reader. Data presented here are means of triplicate experiments, and error bars indicate the SDs. *, statistical significance (*P* < 0.05). (**B**–**E**) Virulence potential of *R. planticola* SCLZS62 in a *G. mellonella* infection model. Survival curves for *G. mellonella* larvae inoculated with 1 × 10^4^ CFU (E), 1 × 10^5^ CFU (**D**), 1 × 10^6^ CFU (**C**), and 1 × 10^7^ CFU (**B**) of each strain are shown. *K. pneumoniae* NTUH-K2044 serves as the hypervirulence-positive control, and *E. coli* MG1655 as the negative control.

Biofilm formation assays showed that the biofilm-forming ability of SCLZS62 was significantly higher than that of *E. coli* MG1655, and comparable to that of *K. pneumoniae* NTUH-K2044 (Fig. 6A). The pathogenic potential of SCLZS62 was determined by *Galleria mellonella* infection testing. Results showed that the survival rates of *G. mellonella* significantly decreased when infected with strain SCLZS62 or NTUH-K2044 in relative to those with MG1655 under various infection concentrations (Fig. 6B–E). These findings suggested that SCLZS62 is a biofilm producer, and potentially virulent. In silico analysis detected 38 virulence genes in *R. planticola* SCLZS62 (Table S4), including gene clusters encoding type 3 fimbriae (*mrkABC*) and type 1 fimbriae (*fimACDEGH*), which contribute to biofilm formation, and adherence to host cells^54^. Also, genes encoding aerobactin (*iutA*) and ent siderophore (*entABCES* and *fepABCG*) for iron acquisition, and *gnd* for serum resistance were identified, which could enhance the ability of the bacteria to survive and colonize within the host^55^. Additionally, several sets of genes encoding type VI secretion system were included, such as *dotU/tssL*,* hcp/tssD*,* icmF/tssM* and *impA/tssA*,* vipB/tssC*,* vasE/tssK,* which play an important role in the invasion and pathogenicity during the infection process of pathogens^56^.

## Conclusions

In this study, we report a carbapenem- and tigecycline-resistant *R. planticola* isolate carrying nine plasmids from hospital sewage. The coexistence of nine plasmids provides great genetic plasticity for further spread of resistance genes, and the fitness of host bacteria to its environment. This isolate contains two types of carbapenemase genes, including one *bla*_KPC-2_ and two *bla*_NDM-1_ genes, which confirms the dissemination of clinically important resistance genes into the environment by sewage discharged from hospitals. The coproduction of multiple determinants for the same resistance is mystifying, while it hints at the important host role of *R. planticola* in the propagation of ARGs. Finally, we identified that this MDR isolate is potentially virulent, which poses a potential public health risk. The occurrence of virulent superbug in the environment should be closely monitored.

## Supplementary Information

Supplementary Information.

## Data Availability

The complete sequences of the chromosome of SCLZS62 and its plasmids have been deposited in GenBank under the Project No. PRJNA757536.
