# Supplementary material for: Genetic and virulence characteristics of a Raoultella planticola isolate resistant to carbapenem and tigecycline
Source: Sci Rep. 2022 Mar 9;12:3858. doi: 10.1038/s41598-022-07778-0 (PMC8907287; doi:10.1038/s41598-022-07778-0)
Supplement: Supplementary file 1 — Supplementary Information. [file 41598_2022_7778_MOESM1_ESM.docx]

**Table S1 Primers used in this study.**

| **Primer name** | **Sequence (5’-3’)** | **Source** |
| --- | --- | --- |
| p2-F | GTCAGTCACACCTTCCCGCGCTT | This study |
| p2-R | GGCAGGGAACTGGTTCTGATGAG |  |
| p5-F | CCCATCTTCGAGAGCTCTTTCTG | This study |
| p5-R | GAGAACCAAAGACAAAGACCTG |  |
| p7-F | CTCGAAGTTGTAGCGAATGGGT | This study |
| p7-R | GGGCTTTTTCTCGCTCCTGGTG |  |
| p8-F | CATTCAAGGGCTTTCTTGCTGC | This study |
| p8-R | ACGACGGCATAGTCATTTGC |  |
| p1-F  p1-R  p3-F  p3-R  p4-F  p4-R  p6-F  p6-R  p9-F  p9-R | GTTACCAGTGGCTGCTGCCAGT  CCCCTGACAAGCATCACGAAAT  TATTTGCGAGCTTTTGCCTTGC  AAGCCGAAGGGTACGAGGACAT  ACCCAATATCCTGCAACTGCTT  TATATCGGCGAGGTTATAGGTC  TTACGTCCTAATAGCAGCAGGAC  CAAAACCTTGTGGTCGTACTGAT  TAGAGATGCTGTACCGCCAGCT  ACACTGGAGTCAGCTGGATACG | This study  This study  This study  This study  This study |
| F1 | tgcctgcaggtcgacgatatcTAAGGCGT  AGTGTAGAAGAGACAACA | This study |
| F2 | tgcctgcaggtcgacgatatcGGTTTCACC  ACATAATCGTCAGC | This study |
| R1 | cggggatcctctagagatatcGATGAGCAC  CACCATGCATATCG | This study |
| R2 | cggggatcctctagagatatcTATGAACGCT  GGATACCATACTGAG | This study |

**Table S2 Detailed information of *R. planticola* strains used in the phylogenetic tree** (last accessed 02 September, 2021)

| **Accession no.** | **Strain name** | **Country** | **Year** | **Origin** |
| --- | --- | --- | --- | --- |
| GCA_000648315.1 | TNT rawlib.basecaller_c6 | Belgium | 2013 | Environment |
| GCA_000735435.1 | ATCC 33531 | USA | 2014 | Environment |
| GCA_000737915.1 | CHB | USA | 2012 | Environment |
| GCA_000783935.2 | FDAARGOS_64 | USA | 2013 | Humans |
| GCA_000949945.1 | GCSL-DIFS-295 | USA | 2012 | Animal |
| GCA_001049875.1 | CMUL058 | France | 2015 | Unknown |
| GCA_001065685.1 | 626_SENT | USA | 2014 | Humans |
| GCA_001663315.1 | INSali127 | Portugal | 2013 | Environment |
| GCA_001663325.1 | INSali133 | Portugal | 2013 | Environment |
| GCA_001903595.4 | CRK0162 | USA | 2013 | Humans |
| GCA_002264145.1 | R3467 | China | 2015 | Humans |
| GCA_002554635.2 | FDAARGOS_283 | USA | 2013 | Humans |
| GCA_002588355.1 | FDAARGOS_428 | Canada | 2015 | Humans |
| GCA_002635135.1 | FDAARGOS_429 | Canada | 2015 | Humans |
| GCA_002761975.1 | GDW02598 | Canada | 2014 | Humans |
| GCA_002906195.1 | GODA | China | 2016 | Humans |
| GCA_003699975.1 | FDAARGOS_430 | Canada | 2015 | Humans |
| GCA_004024155.1 | GEO_47_Down_B | USA | 2017 | Environment |
| GCA_004312145.1 | TUM14065 | Japan | 2014 | Humans |
| GCA_004312285.1 | TUM14072 | Japan | 2013 | Humans |
| GCA_004312325.1 | TUM14074 | Japan | 2014 | Humans |
| GCA_004345285.1 | DSM 2688 | USA | 2019 | Unknown |
| GCA_004365995.1 | 103 | USA | 2019 | Environment |
| GCA_004366295.1 | 103_o | USA | 2019 | Environment |
| GCA_005048625.1 | KS-EMBGN | USA | 2019 | Environment |
| GCA_006757685.1 | M30b | Colombia | 2009 | Environment |
| GCA_008694045.1 | S25 | China | 2017 | Animal |
| GCA_008925805.1 | 7084_4 | USA | 2018 | Humans |
| GCA_010597485.1 | AS012340 | USA | 2014 | Humans |
| GCA_010598615.1 | AS012264 | USA | 2016 | Humans |
| GCA_010598665.1 | AS012263 | USA | 2016 | Humans |
| GCA_011290675.2 | HH15 | Canada | 2010 | Unknown |
| GCA_013422945.1_ | 7084_1 | USA | 2018 | Humans |
| GCA_013422955.1 | 7084_5 | USA | 2018 | Humans |
| GCA_013462275.1 | Rp_CZ180511 | China | 2018 | Humans |
| GCA_014855695.1 | RO13458-2 | Germany | 2017 | Environment |
| GCA_014856235.1 | RO-VA5294443 | Germany | 2015 | Humans |
| GCA_015135635.1 | STW0522-56 | Japan | 2018 | Environment |
| GCA_015893245.1 | hkcpe63 | China | 2014 | Humans |
| GCA_015893725.1 | MISC063 | Australia | 2018 | Animal |
| GCA_015893745.1 | MISC077 | Australia | 2018 | Animal |
| GCA_015893765.1 | MISC062 | Australia | 2018 | Animal |
| GCA_015893805.1 | I089 | Australia | 2001 | Animal |
| GCA_015893825.1 | MISC759 | Australia | 2018 | Animal |
| GCA_015893865.1 | MISC076 | Australia | 2018 | Animal |
| GCA_015893925.1 | I105 | Australia | 2001 | Animal |
| GCA_018170975.1 | 1D-172 | Denmark | 2018 | Humans |
| GCA_018443145.1 | Survcare086 | Germany | 2018 | Humans |
| GCA_018443165.1 | Survcare085 | Germany | 2018 | Humans |
| GCA_019725635.1 | N20E287203-01 | USA | 2020 | Humans |
| GCA_900083755.1 | 2880STDY5682802 | United Kingdom | 2008 | Humans |
| GCA_900455785.1 | NCTC9528 | United Kingdom | 2018 | Unknown |
| GCA_900706865.1 | NCTC12998 | United Kingdom | 2019 | Unknown |
| GCA_901420545.1 | NCTC9155 | United Kingdom | 2019 | Humans |
| GCA_901420695.1 | NCTC9527 | United Kingdom | 2019 | Environment |
| GCA_901420725.1 | NCTC9179 | United Kingdom | 2019 | Humans |
| GCA_902160325.1 | 4928STDY7071414 | United Kingdom | 2019 | Humans |
| GCA_902807195.1 | PBIO703 | Germany | 2020 | Humans |
| This study | SCLZS62 | China | 2019 | Environment |

**Table S3 MIC profiles for *R. planticola* SCLZS62 and transformants**.

| **Strains** | **MIC (μg/ml) ^a^** | | | | | | | | | | |  |
| --- | --- | --- | --- | --- | --- | --- | --- | --- | --- | --- | --- | --- |
|  | **AMK** | **FOS** | **GEN** | **CST** | **MEM** | **ACR** | **CRV** | **CHL** | **CIP** | **CTX** | **TGC** | |
| *R. planticola* SCLZS62 | >512 | 512 | 512 | 4 | 512 | - | - | 4 | 8 | >512 | 8 | |
| *E. coli* DH5α | 1 | ≤0.5 | ≤0.5 | 1 | ≤0.5 | 8 | 1 | 4 | ≤0.5 | ≤0.5 | ≤0.5 | |
| DH5α/pUC19 | ≤0.5 | ≤0.5 | ≤0.5 | 2 | ≤0.5 | 8 | 1 | 4 | ≤0.5 | ≤0.5 | ≤0.5 | |
| DH5α/pMD19-*baeSR*-*tmexAB-toprM* | 4 | ≤0.5 | ≤0.5 | ≤0.5 | ≤0.5 | 8 | 2 | 2 | ≤0.5 | ≤0.5 | ≤0.5 | |
| DH5α/pMD19-*tmexAB-toprM* | 2 | 1 | 1 | ≤0.5 | ≤0.5 | 8 | 2 | 1 | ≤0.5 | ≤0.5 | ≤0.5 | |
| DH5α/pMD19-*baeSR*-*tmexAB* | 8 | ≤0.5 | 4 | ≤0.5 | ≤0.5 | 8 | 1 | ≤0.5 | ≤0.5 | ≤0.5 | ≤0.5 | |
| *E. coli* ATCC25922 | 4 | 2 | 2 | 4 | ≤0.5 | 8 | 8 | 4 | ≤0.5 | ≤0.5 | ≤0.5 | |

^a^ AMK, amikacin; FOS, fosfomycin; GEN, gentamicin; CST, colistin; MEM, meropenem; ACR, acriflavine; CRV, Crystal violet; CHL, chloramphenicol; CIP, ciprofloxacin; CTX, cefotaxime; TGC, tigecycline.

**Table S4** **Virulence gene profiles of *R. planticola* SCLZS62.**

| **Virulence gene** | **Identity (100%)** | **Query / Template length** | **VFclass** | **Accession number** |
| --- | --- | --- | --- | --- |
| acrB | 86 | 3147/3147 | Secretion system | [VFG049144](http://www.mgc.ac.cn/cgi-bin/VFs/gene.cgi?GeneID=VFG049144) |
| acrA | 85 | 1194/1194 | Secretion system | [VFG049133](http://www.mgc.ac.cn/cgi-bin/VFs/gene.cgi?GeneID=VFG049133) |
| icmF/tssM | 83 | 1879/3456 | Secretion system | [VFG048755](http://www.mgc.ac.cn/cgi-bin/VFs/gene.cgi?GeneID=VFG048755) |
| tssF | 84 | 1131/1755 | Secretion system | [VFG048771](http://www.mgc.ac.cn/cgi-bin/VFs/gene.cgi?GeneID=VFG048771) |
| ompA | 88 | 585/1041 | Secretion system | [VFG001443](http://www.mgc.ac.cn/cgi-bin/VFs/gene.cgi?GeneID=VFG001443) |
| vipB/tssC | 83 | 872/1545 | Secretion system | [VFG048643](http://www.mgc.ac.cn/cgi-bin/VFs/gene.cgi?GeneID=VFG048643) |
| hcp/tssD | 89 | 488/492 | Secretion system | [VFG048687](http://www.mgc.ac.cn/cgi-bin/VFs/gene.cgi?GeneID=VFG048687) |
| impA/tssA | 81 | 929/1599 | Secretion system | [VFG048762](http://www.mgc.ac.cn/cgi-bin/VFs/gene.cgi?GeneID=VFG048762) |
| tssG | 81 | 811/1086 | Secretion system | [VFG048779](http://www.mgc.ac.cn/cgi-bin/VFs/gene.cgi?GeneID=VFG048779) |
| vasE/tssK | 80 | 965/1344 | Secretion system | [VFG048654](http://www.mgc.ac.cn/cgi-bin/VFs/gene.cgi?GeneID=VFG048654) |
| dotU/tssL | 83 | 488/690 | Secretion system | [VFG048665](http://www.mgc.ac.cn/cgi-bin/VFs/gene.cgi?GeneID=VFG048665) |
| gndA | 84 | 1407/1407 | Serum resistance | [VFG048830](http://www.mgc.ac.cn/cgi-bin/VFs/gene.cgi?GeneID=VFG048830) |
| fimD | 83 | 2175/2613 | Adherence | [VFG048277](http://www.mgc.ac.cn/cgi-bin/VFs/gene.cgi?GeneID=VFG048277) |
| mrkC | 83 | 1919/2487 | Adherence | [VFG043625](http://www.mgc.ac.cn/cgi-bin/VFs/gene.cgi?GeneID=VFG043625) |
| Ugd | 84 | 1163/1167 | Adherence | [VFG048797](http://www.mgc.ac.cn/cgi-bin/VFs/gene.cgi?GeneID=VFG048797) |
| mrkB | 86 | 679/702 | Adherence | [VFG043626](http://www.mgc.ac.cn/cgi-bin/VFs/gene.cgi?GeneID=VFG043626) |
| fimH | 83 | 859/906 | Adherence | [VFG048307](http://www.mgc.ac.cn/cgi-bin/VFs/gene.cgi?GeneID=VFG048307) |
| Wzi | 81 | 1067/1434 | Adherence | [VFG048980](http://www.mgc.ac.cn/cgi-bin/VFs/gene.cgi?GeneID=VFG048980) |
| mrkA | 85 | 609/609 | Adherence | [VFG043627](http://www.mgc.ac.cn/cgi-bin/VFs/gene.cgi?GeneID=VFG043627) |
| fimC | 84 | 476/726 | Adherence | [VFG048267](http://www.mgc.ac.cn/cgi-bin/VFs/gene.cgi?GeneID=VFG048267) |
| galF | 84 | 483/891 | Adherence | [VFG048990](http://www.mgc.ac.cn/cgi-bin/VFs/gene.cgi?GeneID=VFG048990) |
| fimE | 81 | 560/609 | Adherence | [VFG048237](http://www.mgc.ac.cn/cgi-bin/VFs/gene.cgi?GeneID=VFG048237) |
| fimA | 83 | 545/549 | Adherence | [VFG048247](http://www.mgc.ac.cn/cgi-bin/VFs/gene.cgi?GeneID=VFG048247) |
| fimG | 80 | 439/486 | Adherence | VFG048297 |
| fepA | 86 | 1577/2229 | Iron uptake | [VFG048518](http://www.mgc.ac.cn/cgi-bin/VFs/gene.cgi?GeneID=VFG048518) |
| fepB | 80 | 916/960 | Iron uptake | [VFG048449](http://www.mgc.ac.cn/cgi-bin/VFs/gene.cgi?GeneID=VFG048449) |
| entA | 88 | 528/747 | Iron uptake | [VFG048409](http://www.mgc.ac.cn/cgi-bin/VFs/gene.cgi?GeneID=VFG048409) |
| entE | 87 | 1086/1608 | Iron uptake | [VFG048429](http://www.mgc.ac.cn/cgi-bin/VFs/gene.cgi?GeneID=VFG048429) |
| Fur | 87 | 341/453 | Iron uptake | [VFG000478](http://www.mgc.ac.cn/cgi-bin/VFs/gene.cgi?GeneID=VFG000478) |
| entS | 85 | 1217/1242 | Iron uptake | [VFG048459](http://www.mgc.ac.cn/cgi-bin/VFs/gene.cgi?GeneID=VFG048459) |
| entB | 88 | 852/852 | Iron uptake | [VFG048419](http://www.mgc.ac.cn/cgi-bin/VFs/gene.cgi?GeneID=VFG048419) |
| entC | 86 | 830/1176 | Iron uptake | [VFG048439](http://www.mgc.ac.cn/cgi-bin/VFs/gene.cgi?GeneID=VFG048439) |
| fepG | 85 | 862/993 | Iron uptake | [VFG048478](http://www.mgc.ac.cn/cgi-bin/VFs/gene.cgi?GeneID=VFG048478) |
| fepC | 85 | 779/795 | Iron uptake | [VFG048488](http://www.mgc.ac.cn/cgi-bin/VFs/gene.cgi?GeneID=VFG048488) |
| iutA | 83 | 2180/2190 | Iron uptake | [VFG048620](http://www.mgc.ac.cn/cgi-bin/VFs/gene.cgi?GeneID=VFG048620) |
| rcsB | 88 | 651/651 | Regulation | VFG049018 |
| phoP | 79 | 594/675 | Regulation | [VFG000475](http://www.mgc.ac.cn/cgi-bin/VFs/gene.cgi?GeneID=VFG000475) |
| rpoS | 86 | 989/993 | Regulation | [VFG000477](http://www.mgc.ac.cn/cgi-bin/VFs/gene.cgi?GeneID=VFG000477) |


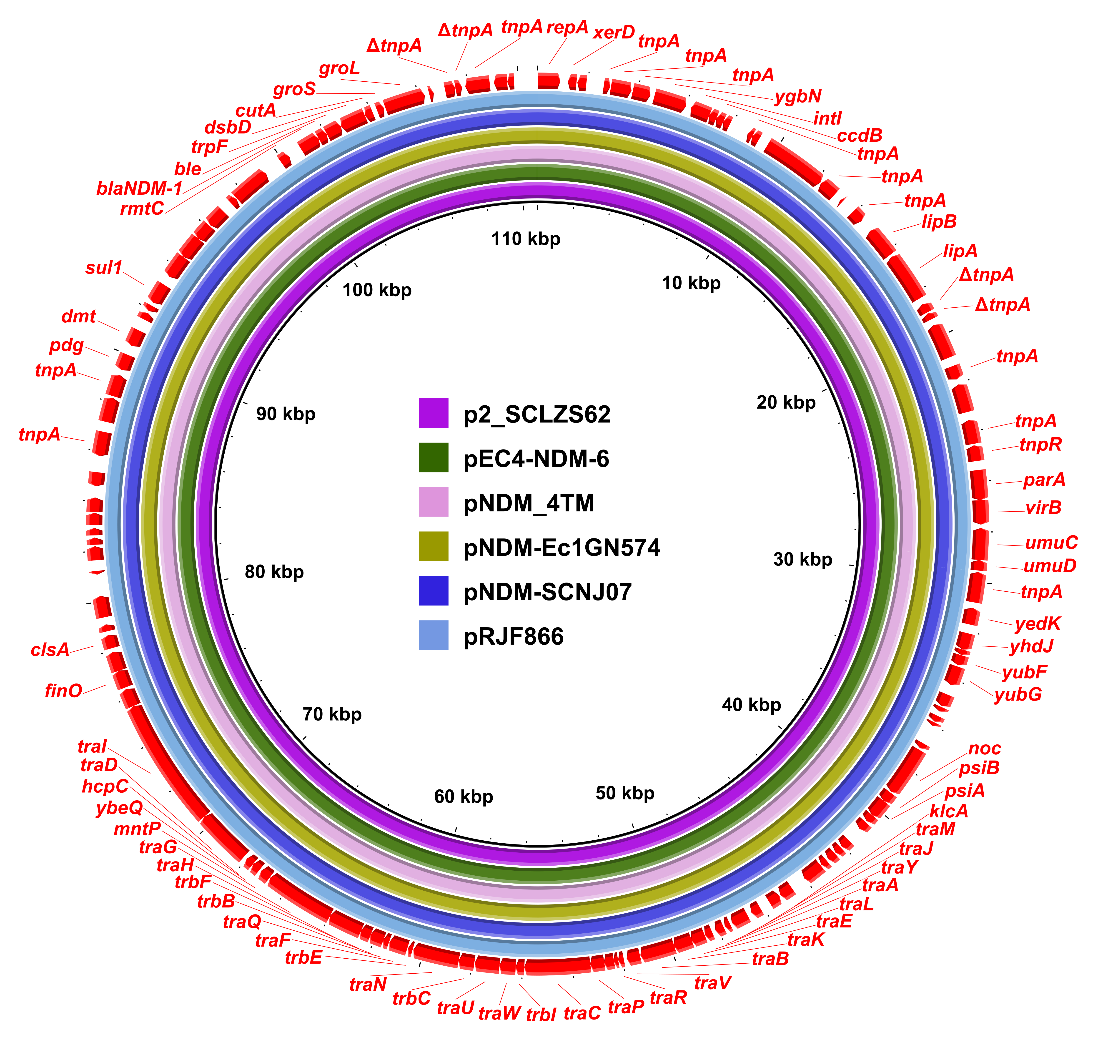


**Figure S1 Circular comparison of p2_SCLZS62 with closely related plasmids.** The arrows indicate deduced ORFs and their orientations. The alignment was performed using BRIG and p2_ SCLZS62 was used as a reference. GenBank accession numbers for the plasmids are KC887916 (pEC4-NDM-6), MF042352 (pNDM_4TM), KJ812998 (pNDM-Ec1GN574), MK933278 (pNDM-SCNJ07) and KF732966 (pRJF866).


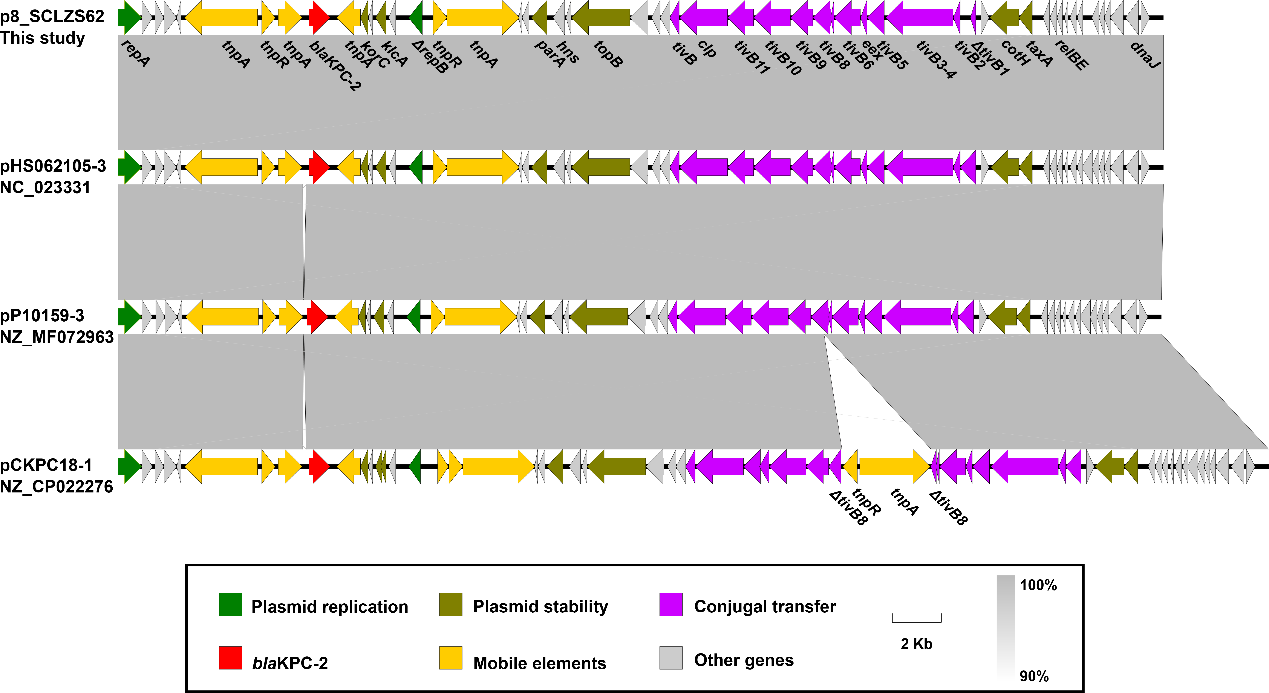


**Figure S2 Comparative analysis of p8_SCLZS62 and closely related plasmids.** Genes are denoted by arrows and are colored based on their functional classification. Grey shading denotes regions of shared homology ranging from 90% to 100%. Δ represents truncated genes or mobile genetic elements.
